# Glutaraldehyde Cross-Linking of Immobilized Thermophilic Esterase on Hydrophobic Macroporous Resin for Application in Poly(ε-caprolactone) Synthesis

**DOI:** 10.3390/molecules19079838

**Published:** 2014-07-08

**Authors:** Min Wang, Hui Shi, Di Wu, Haobo Han, Jianxu Zhang, Zhen Xing, Shuang Wang, Quanshun Li

**Affiliations:** 1 Department of Colorectal and Anal Surgery, The Second Hospital, Jilin University, Changchun 130041, China; E-Mail: wangmin20060513@hotmail.com; 2 Key Laboratory for Molecular Enzymology and Engineering of Ministry of Education, School of Life Sciences, Jilin University, Changchun 130012, China; E-Mails: shihui13@mails.jlu.edu.cn (H.S.); Michaelwu_1990@live.cn (D.W.); hanhb1310@mails.jlu.edu.cn (H.H.); longde.guxiang@163.com (J.Z.); silence__xz@163.com (Z.X.); 3 Department of Dermatology, The Second Hospital, Jilin University, Changchun 130041, China

**Keywords:** immobilized enzyme, cross linking, thermophilic esterase, ring-opening polymerization, ε-caprolactone

## Abstract

The immobilized thermophilic esterase from *Archaeoglobus fulgidus* was successfully constructed through the glutaraldehyde-mediated covalent coupling after its physical adsorption on a hydrophobic macroporous resin, Sepabeads EC-OD. Through 0.05% glutaraldehyde treatment, the prevention of enzyme leaching and the maintenance of catalytic activity could be simultaneously realized. Using the enzymatic ring-opening polymerization of ε-caprolactone as a model, effects of organic solvents and reaction temperature on the monomer conversion and product molecular weight were systematically investigated. After the optimization of reaction conditions, products were obtained with 100% monomer conversion and *M*_n_ values lower than 1010 g/mol. Furthermore, the cross‑linked immobilized thermophilic esterase exhibited an excellent operational stability, with monomer conversion values exceeding 90% over the course of 12 batch reactions, still more than 80% after 16 batch reactions.

## 1. Introduction

In the past two decades, enzymatic polymerization, especially lipase/esterase-catalyzed synthesis of polyesters, has been greatly developed and considered as an important route for polymer synthesis [1,2,3,4,5,6,7]. Compared with conventional chemical routes, enzymatic polymerization has many advantages, including: (1) mild reaction conditions, (2) high control of enantio-, chemo-, and regio-selectivity, (3) high catalytic activity toward macrocyclic lactones (difficult to polymerize via chemical catalysis), and (4) no trace residues of metallic catalysts, which will be favorable for solving the toxicity issue in biomedical applications. 

Among the products prepared through enzymatic polymerization, poly(ε-caprolactone) (PCL) is the most widely investigated one, as it is an important type of biodegradable and biocompatible polymer used in various biomedical applications [8], e.g., resorbable implant materials for tissue engineering and vehicles for drug/gene delivery. To date, several lipases or esterases have been employed as catalysts for PCL synthesis, such as *Candida antarctica* lipase B, *Pseudomonas cepacia* lipase and *Humicola insolens* cutinase [9,10,11]. Compared with these mesophilic enzymes, enzymes from thermophiles exhibited better potential in the preparation of PCL, due to their excellent stability against high temperature and organic solvents [12]. In our previous reports, thermophilic lipase FNL from *Fervidobacterium nodosum* and esterase AFEST from *Archaeoglobus fulgidus* have been successfully explored with excellent activity for PCL synthesis (*ca.* 100% monomer conversion), and products with low number-average molecular weight (*M*_n_ < 3000 g/mol) were usually obtained [13,14]. Besides using thermophilic enzymes as catalysts, another method for improving the catalytic activity and stability under harsh reaction conditions is to construct immobilized enzymes, particularly for realizing the biocatalytic synthesis of polyesters at an industrial scale [15,16,17]. Meanwhile, the operational stability for repeated use could be improved, thereby decreasing the costs in the enzymatic synthesis of PCL [18,19]. Chen *et al.* once employed macroporous methyl methacrylate, polystyrene resins and epoxy-activated micro- and nanobeads as carriers to prepare immobilized *C. antarctica* lipase B, and successfully applied them in the polyester synthesis [20,21,22]. Poojari *et al.* constructed an immobilized *C. antarctica* lipase B through physical adsorption onto macroporous acrylic resin, and this immobilized enzyme could efficiently catalyze the PCL synthesis, with weight‑average molecular weight (*M*_w_) of *ca.* 50,000 g/mol [23]. Similarly, our research group adsorbed AFEST to the hydrophobic macroporous resin Octadecyl-Sepabeads EC-OD and used it for the synthesis of PCL [24]. As the support is highly hydrophobic, it could adsorb the open form of lipase (fixed on the interfacially adsorbed derivative), and the immobilized lipase exhibited higher activity and stability [25]. However, in our research, the immobilized enzyme possessed only 36% of the original synthetic activity after five reuses, probably due to the loss of adsorbed enzyme from the support during consecutive reactions [24,26]. In fact, all these immobilized enzymes are used with the problem of limited operational stability, and thus developing more effective biocatalysts through chemical crosslinkages or protein modification will be of great significance for realizing enzymatic polymerization at an industrial scale in future. Glutaraldehyde is a reagent widely used to modify proteins, mainly involving primary amino groups of proteins [27]. In addition, it is a very effective crosslinker to produce intermolecular crosslinking of enzymes, almost in any type of supports mainly if the enzyme molecules are closely packed together [28,29]. 

**Scheme 1:**
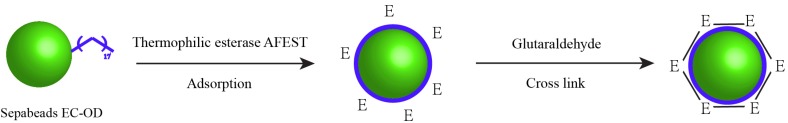
Schematic representation for constructing the cross-linked immobilized thermophilic esterase via glutaraldehyde chemistry.

In the present study, a cross-linked immobilized thermophilic esterase was prepared through the covalent coupling mediated by glutaraldehyde treatment after the physical adsorption of thermophilic esterase AFEST on a hydrophobic macroporous resin Sepabeads EC-OD (Scheme 1), and then its application in PCL synthesis was systematically assessed, especially for its operational stability. 

## 2. Results and Discussion

### 2.1. Preparation of Cross-Linked Immobilized Thermophilic Esterase

**Figure 1:**
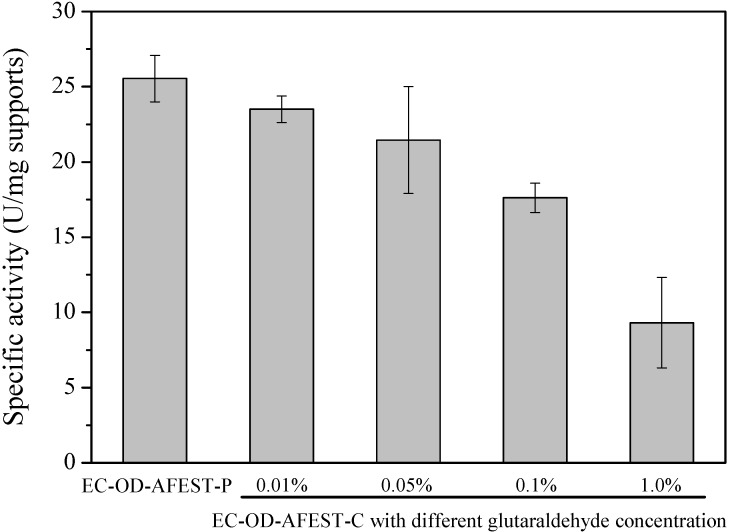
Effect of glutaraldehyde concentration on the specific activity of immobilized enzymes.

Using macroporous resin Sepabeads EC-OD as carrier, the cross-linked immobilized thermophilic esterase was prepared via a two-step route, as shown in Scheme 1. The first step is to adsorb enzymes on the macroporous resins and the second step is to crosslink the adsorbed enzymes via glutaraldehyde chemistry, resulting in a cross-linked immobilized thermophilic esterase on the surface of macroporous resins. As reported previously [13], the thermophilic esterase AFEST was partially purified, with an active protein content of 92% (w/w) in the enzyme preparation, and the specific activity of lyophilized enzyme was approximately 168 U/mg toward *p*-nitrophenyl caprylate at 80 °C. Through the physical adsorption for 24 h, the adsorption efficiency was measured to be 76%, and thus the loading of AFEST on supports was calculated as 152 mg AFEST/g supports [24]. After the physical adsorption, cross‑linking of enzymes on the surface of supports was then conducted through the treatment with different concentrations of glutaraldehyde. Generally, two key factors should be considered in the glutaraldehyde treatment process: the prevention of enzyme leaching and the maintenance of catalytic activity [30]. During the glutaraldehyde treatment, almost no protein leaching was detected, indicating 0.01% glutaraldehyde was just enough to prevent the enzymes leaching out. For the specific activity, the glutaraldehyde concentration has a great influence on the enzymatic activity. As shown in Figure 1, with the increasing of glutaraldehyde concentration, the specific activity would be decreased, with only 9.31 U/mg supports after 1.0% glutaraldehyde treatment. Here, both 0.01% and 0.05% glutaraldehyde treatment could produce the immobilized enzymes with relatively higher catalytic activity. 

### 2.2. Optimization of Reaction Conditions for Enzymatic Polymerization

**Figure 2:**
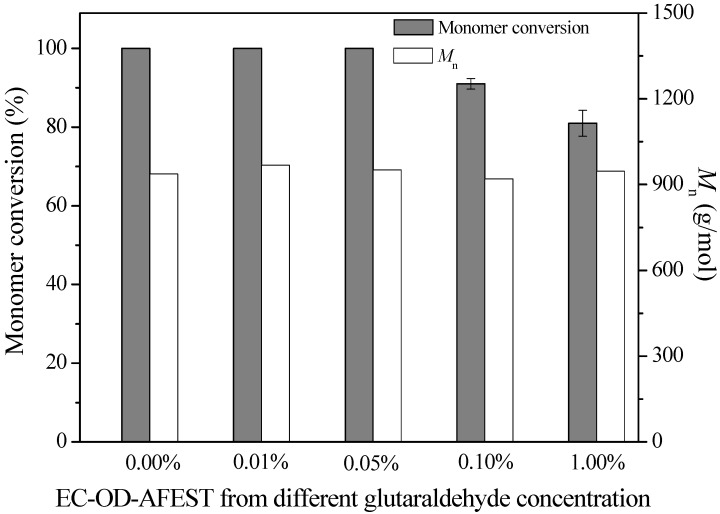
Enzymatic ring-opening polymerization of ε-caprolactone using different immobilized enzymes. The reactions were carried out using 60 mg immobilized enzymes, 200 μL ε-caprolactone and 600 μL toluene at 80 °C for 72 h.

In lipase/esterase-catalyzed ring-opening polymerization of ε-caprolactone, reaction conditions play a very important role for improving monomer conversion and product molecular weight. First, we evaluated the effects of glutaraldehyde treatment on the ring-opening polymerization of ε‑caprolactone. The reactions were carried out using 60 mg immobilized enzymes, 200 μL ε‑caprolactone and 600 μL toluene at 80 °C for 72 h. As shown in Figure 2, using immobilized enzyme prepared from 0.01% and 0.05% glutaraldehyde treatment, the monomer conversion exhibited no changes due to the intrinsic high activity of samples. With the increasing concentration of glutaraldehyde (0.10% to 1.00%), the monomer conversion values would be decreased, but they were still in a high level (>80%). Meanwhile, the glutaraldehyde treatment exhibited almost no obvious influences on the *M*_n_ values (920–970 g/mol). As 0.01% glutaraldehyde treatment might be unfavorable for the prevention of enzyme leaching in organic solvents, 0.05% glutaraldehyde treatment was employed to construct the cross-linked immobilized thermophilic esterase, and then used in the further optimization of reaction conditions. Moreover, in the present study, PCL was prepared with low molecular weight, and was potential to be widely used as soft segment for polyurethanes or carriers for localized drug delivery, similar to low viscosity poly(trimethylene carbonate) [31]. 

It has been generally accepted that the reaction medium plays a crucial role in determining the stability of the biocatalyst and in the partitioning of substrates and products between the solvent and the biocatalyst in non-aqueous biocatalytic systems [32]. Table 1 showed the effect of organic solvents with different Log *P* values on monomer conversion and *M*_n_ at 70 °C for 72 h. Similar to our previous reports [13,14,24], hydrophobic solvents (toluene, cyclohexane and *n*-hexane) were favorable for the reaction, with high monomer conversion (100%) and *M*_n_ values (>950 g/mol). The phenomenon was caused by the fact that the hydrophobic solvents could efficiently keep the essential water layer surrounding the enzyme molecules and did not disrupt the functional structure of enzymes [33]. As the products were not dissolved in the solvents cyclohexane and *n*-hexane, these solvents were not suitable for the purification of PCL, and thus toluene was usually selected as the optimal reaction medium in enzymatic PCL synthesis. In addition, in solvent-free system, products could be obtained with 87% monomer conversion and *M*_n_ of 920 g/mol, providing a much greener route for polyester synthesis. 

**Table 1:** Effects of organic solvents on monomer conversion and product molecular weight *M*_n_.

Solvent	Log *P*	Monomer Conversion (%)	*M*_n_ (g/mol)	PDI
1,4-Dioxane	−1.10	n.d.	n.d.	n.d.
Acetone	−0.23	70	835	1.09
Tetrahydrofuran	0.49	76	890	1.09
Dichloromethane	0.93	n.d.	n.d.	n.d.
Chloroform	2.00	83	914	1.12
Toluene	2.50	100	952	1.17
Cyclohexane	3.09	100	1015	1.24
*n*-Hexane	3.50	100	1070	1.24
Solvent-free	—	87	920	1.10

n.d.: not determined.

**Figure 3:**
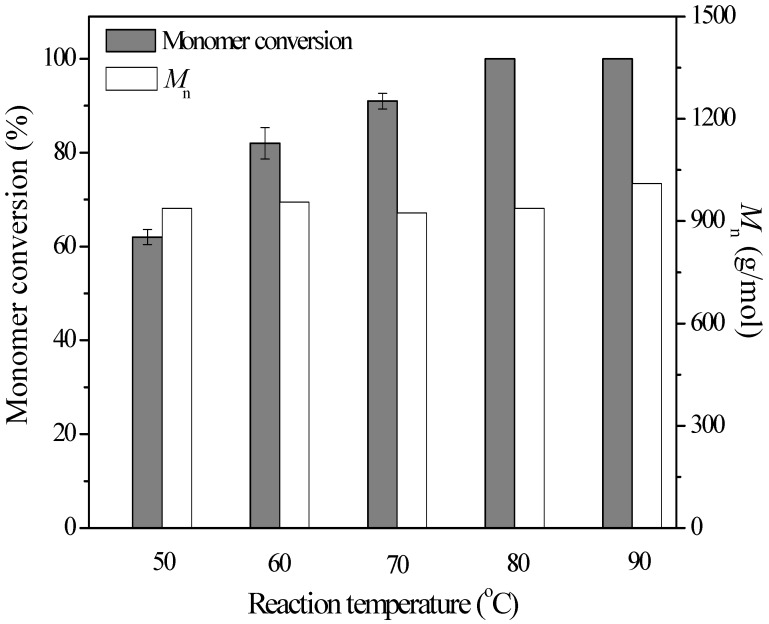
Immobilized enzyme-catalyzed ring-opening polymerization of ε-caprolactone at different temperatures. The reactions were carried out using 60 mg immobilized enzymes, 200 μL ε-caprolactone and 600 μL toluene for 72 h.

The effect of temperature on monomer conversion and product molecular weight was investigated at different temperatures in toluene for 72 h. As shown in Figure 3, it was obvious that high temperature was favorable for the reactions. After 72 h, the monomer conversion increased from 62% at 50 °C to 100% at 80 °C or 90 °C. However, different from free enzyme [13], the temperature exhibited no effects on the product molecular weight, in the range of 920–1010 g/mol. As there were no significant differences between reactions at 80 and 90 °C, 80 °C was selected as the optimal reaction temperature for the immobilized enzyme-catalyzed PCL synthesis. Thus, both the intrinsic high thermostability of AFEST and stabilization of immobilization technique made the immobilized enzyme more suitable for polyester synthesis at a larger scale, and even for realizing the simultaneous, single-step chemoenzymatic synthesis of novel polymers. 

### 2.3. Operational Stability

**Figure 4:**
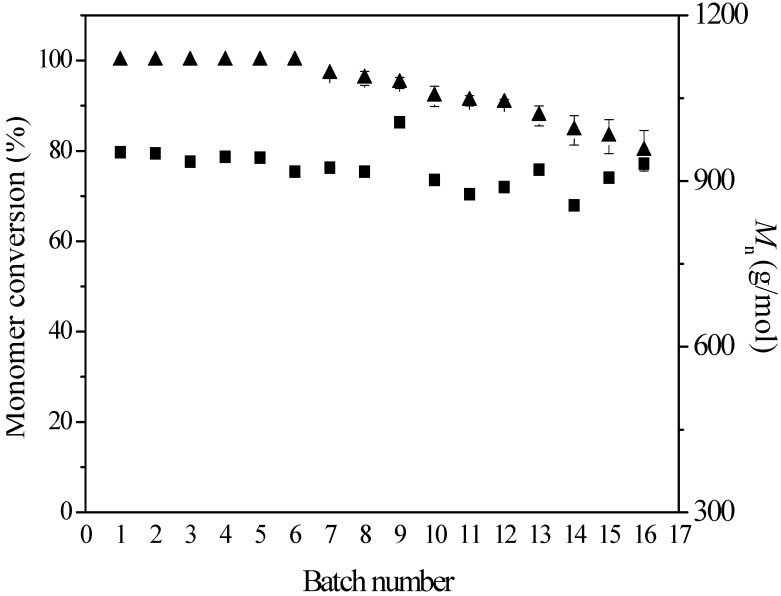
Monomer conversion and *M*_n_ values for a series of consecutive batch reactions conducted using 60 mg immobilized enzyme as catalyst; on completion of one reaction, the catalyst was washed and recycled for use in the next. The reactions were carried out using 60 mg immobilized enzymes, 200 μL ε-caprolactone and 600 μL toluene at 80 °C for 72 h.

To test the operational stability of immobilized enzyme EC-OD-AFEST-C, a series of consecutive ring-opening polymerization reactions were performed in toluene at 80 °C for 72 h using 60 mg of immobilized enzyme as a catalyst. As shown in Figure 4, the immobilized enzymes exhibited good operational stability, with monomer conversion value more than 90% after 12 batch reactions, even still exceeding 80% after 16 batch reactions. 

Meanwhile, the *M*_n_ values were kept at a relatively constant value (850–1010 g/mol). Nevertheless, using immobilized enzyme EC-OD-AFEST-P prepared through physical adsorption as a catalyst, monomer conversion values showed a distinct declining tendency in the five repeated uses, from 100% (1st batch) to 36% (5th batch) [24]. Therefore, the combination of physical adsorption and glutaraldehyde-mediated crosslinking could be used to construct immobilized enzymes with better operational stability, avoiding the loss of adsorbed enzymes from the supports during the reactions. 

## 3. Experimental Section

### 3.1. Materials

The recombinant *E. coli* strain harboring the thermophilic esterase gene AF1716 from *A. fulgidus* was kindly provided by Dr. Giuseppe Manco (Istituto di Biochimica delle Proteine, Napoli, Italy). Octadecyl-Sepabeads EC-OD was kindly provided by Resindion S.R.L. (Mitsubishi Chemical Co., Binasco, Italy). ε-Caprolactone was purchased from Aldrich (Milwaukee, WI, USA) and used as received. Organic solvents of analytic grade were purchased from Beijing Chemical Co. (Beijing, China), and dried over 4 Å molecular sieves (Tianjin Chemical Co., Tianjin, China) before use. Yeast extract and tryptone was purchased from Oxide Ltd. (Basingstoke, UK). Isopropyl β-D-thiogalactopyranoside, ampicillin and *p*-nitrophenyl caprylate were purchased from Sigma (St. Louis, MO, USA). All other reagents were used without further purification.

### 3.2. Preparation of Cross-Linked Immobilized Thermophilic Esterase

The thermophilic esterase AFEST was purified from the recombinant *E. coli* BL21 strain harboring the gene AF1716 from *A. fulgidus* according to the previous reports [13,24,34]. The esterase activity was monitored by measuring the amount of *p*-nitrophenyl using *p*-nitrophenyl caprylate as substrate at 80 °C [35], and one unit of activity was defined as the protein amount releasing 1 μmol *p*-nitrophenyl in one minute. After the purification of thermophilic esterase AFEST, the cross-linked immobilized thermophilic esterase was prepared. Briefly, Sepabeads EC-OD (0.5 g) was washed with ethanol, distilled water and 50 mM phosphate buffer (pH 8.0) twice, and then mixed with 100 mL enzyme solution (1 mg/mL, 50 mM phosphate buffer (pH 8.0)). The vial was incubated at room temperature in a shaking condition (200 rpm) for 24 h. The samples were filtrated, washed with 50 mM phosphate buffer (pH 8.0), and incubated in the same buffer containing various glutaraldehyde concentration (0, 0.01, 0.05, 0.1 and 1% w/w) at 200 rpm for an additional 30 min. After the glutaraldehyde treatment, the cross-linked immobilized enzyme EC-OD-AFEST-C was obtained through filtration, washing with 50 mM phosphate buffer (pH 8.0) and lyophilization. The immobilized enzyme prepared via physical adsorption was named as EC-OD-AFEST-P. The immobilization process was monitored by measuring the enzymatic activity and protein concentration in the supernatant. The enzymatic activity reported was the average of three experiments, and the error represented the standard deviation of those trials. The adsorption efficiency (%) was defined as the ratio of adsorption activity to total activity in the supernatant. The loading of AFEST on the supports (mg AFEST/g supports) was calculated, based on the adsorption efficiency and initial amount of enzyme. 

### 3.3. Enzymatic Ring-Opening Polymerization of ε-caprolactone

The immobilized enzyme was first dried in a desiccator overnight, and then employed in the ring‑opening polymerization of ε-caprolactone. Briefly, ε-caprolactone (200 μL), organic solvent (600 μL) and internal standard (ethylbenzene, 50 μL) were mixed together in a dried screwed vial. The reaction was initiated after adding a quantity of immobilized enzymes into the system. After stirring (180 rpm) for regular intervals, an aliquot of reaction mixture (10 μL) was taken via a syringe, diluted with dichloromethane (100 μL) and then analyzed by gas chromatography (GC). After the reaction, dichloromethane was added into the system, and the immobilized enzymes were removed. Dichloromethane was then removed through evaporation under reduced pressure, and the remaining viscous sample was precipitated in cold methanol (−20 °C). The white precipitate was obtained through centrifugation (8000 rpm) for 15 min, and then dried in a vacuum oven. The polymer structure was characterized by ^1^H-NMR on an AVANCE DMX 500 spectrometer (Bruker Analytische Messtechnik GmbH, Rheinstetten, Germany) at 500 MHz relative to tetramethylsilane as follows: 1.39 (m, -COCH_2_CH_2_C*H_2_*-), 1.66 (m, -COCH_2_C*H_2_*CH_2_C*H_2_*CH_2_O-), 2.31 (t, -COC*H_2_*-), 4.06 (t, -C*H_2_*O-), 3.65 (t, -C*H_2_*OH end group), 2.36 ppm attributable to -COC*H_2_*- of cyclic oligomers. 

### 3.4. Determination of Monomer Conversion and Product Molecular Weight

The monomer conversion was measured through GC analysis on a Shimadzu 2014 gas chromatograph (Shimadzu, Tokyo, Japan) equipped with an Rtx-1 capillary column (30 m × 0.25 mm × 0.25 μm) and a hydrogen flame ionization detector. The temperature program for detection was set as follows: injection pool of 200 °C, detector of 240 °C, and column (70 °C for 2 min to 140 °C for 2 min at a rate of 10 °C/min). Nitrogen was used as the carrier gas. The monomer conversion values were the average of triplicate measurements. For determining the *M*_n_, *M*_w_ and PDI (*M*_w_/*M*_n_) values of products, gel permeation chromatography (GPC) was conducted using a Shimadzu HPLC system with tetrahydrofuran as the eluent at a flow rate of 1.0 mL/min. The GPC system was equipped with a refractive index detector and Shim-pack GPC-804 and GPC-8025 ultrastyragel columns in series. The polystyrene of narrow molecular weight distribution was used as standards to calibrate the GPC system. The sample concentration used in GPC analysis was 0.3% (w/v). The injection volumes for GC and GPC analysis were 1.0 and 20 μL, respectively. 

### 3.5. Operational Stability Analysis

After the ring-opening polymerization of ε-caprolactone at 80 °C for 72 h, the immobilized enzyme was recovered by filtration and washed with dichloromethane. Then the recovered enzyme was employed in repeated reuse as catalyst under the same conditions for evaluating its operational stability. 

## 4. Conclusions

In this paper, the immobilized thermophilic esterse from the archaeon *A. fulgidus* was prepared combining physical adsorption and sequential glutaraldehyde-mediated covalent coupling, using hydrophobic macroporous resin Sepabeads EC-OD as a carrier. The immobilized enzyme was then successfully applied in the PCL synthesis, and exhibited excellent thermostability and operational stability. Thus, it is of great potential for the mild, metal-free synthesis of polyesters. The construction of immobilized enzymes with better operational stability and their application in polyester synthesis at a larger scale are still underway in our laboratory. 
